# Instrumental Mechanoreceptoric Palpation in Gastrointestinal Surgery

**DOI:** 10.1155/2017/6481856

**Published:** 2017-12-31

**Authors:** Rozaliia F. Solodova, Vladimir V. Galatenko, Eldar R. Nakashidze, Sergey G. Shapovalyants, Igor L. Andreytsev, Mikhail E. Sokolov, Vladimir E. Podolskii

**Affiliations:** ^1^Faculty of Mechanics and Mathematics, Lomonosov Moscow State University, Moscow, Russia; ^2^1st Surgery Department, Clinical Hospital No. 31, Moscow, Russia; ^3^Department of Hospital Surgery No. 2, Therapeutic Faculty, N.I. Pirogov Russian National Research Medical University, Moscow, Russia

## Abstract

**Background and Aims:**

Small gastric or colorectal tumours can be visually undetectable during laparoscopic surgeries, and available methods still do not provide a 100% localisation rate. Thus, new methods for further improvements in tumour localisation are highly desirable. In this study, we evaluated the usage of the Medical Tactile Endosurgical Complex (MTEC) in gastrointestinal surgery for localisation of tumours. The MTEC provides the possibility of instrumental mechanoreceptoric palpation, which serves as an analogue of conventional manual palpation.

**Methods:**

Ninety-six elective surgeries were performed, including 48 open surgeries, 43 laparoscopies, and 5 robot-assisted surgeries. The 20 mm version of the MTEC tactile mechanoreceptor was used in open surgeries, and the 10 mm version in laparoscopic and robot-assisted surgeries.

**Results:**

The mean time of instrumental mechanoreceptoric palpation was 3 minutes 12 seconds for open surgeries, which constituted the early stage of the learning curve, and 3 minutes 34 seconds for laparoscopic surgeries. No side effects or postoperative complications related to instrumental mechanoreceptoric palpation were observed, and this procedure provided data sufficient for tumour localisation in more than 95% of cases.

**Conclusion:**

Instrumental mechanoreceptoric palpation performed using MTEC is a simple, safe, and reliable method for tumour localisation in gastrointestinal laparoscopic surgery.

## 1. Introduction

Colorectal and gastric cancers are major causes of morbidity and mortality globally, comprising 9.7% and 6.8% of all cancers, respectively [1]. Specifically, colorectal cancer is the third most commonly diagnosed cancer [1, 2], and gastric cancer is the third mostly common cause of cancer death [1, 3]. Minimally invasive techniques for surgical treatment of these malignancies are now widespread and provide faster recovery, reduction of postoperative pain, and better cosmetic results [4–6]. However, small gastric or colorectal tumours that have not invaded the serosa can be visually undetectable during surgery [7, 8]. Precise localisation of such tumours in open surgeries is performed by palpation, especially in cases of intraluminal malignancies without surface invasion. In laparoscopic surgeries, the haptic feedback from the instrument is frequently insufficient, and localisation of a colonic lesion based solely on colonoscopy is also difficult for such tumours [8, 9]. Thus, additional methods including preoperative CT colonography, tattooing, titanium clips, double contrast barium, intraoperative gastroscopy, or ultrasound examination are used for tumour localisation [7–11]. However, achievement of a 100% localisation rate still evades surgeons [11, 12]. Furthermore, these methods have certain limitations and possible complications. For tattooing most complications are related to transmural injection, and the incidence of invisible lesions (resulting mainly from superficial injection or an injection into the mesenteric side) reaches 15% [12, 13]. Intraoperative ultrasonography or endoscopy requires a specialist at the time of surgery. Insufflation of gas during intraoperative gastroscopy or colonoscopy leads to bowel distention, which reduces the working space available to the surgeon [14].

Thus, new methods and techniques for further improvements and optimisation of localisation of small colorectal and gastric tumours during minimally invasive surgeries are highly desirable. The natural approach is to perform localisation by instrumental mechanoreceptoric palpation, which is applicable in laparoscopic surgery and serves as an analogue of conventional manual palpation in open surgery. To the best of our knowledge, the only currently available commercial device for intraoperative instrumental mechanoreceptoric palpation is the Medical Tactile Endosurgical Complex (MTEC). Results of MTEC usage in thoracoscopic surgery have been reported previously [15]. In this research, we evaluated the usage of MTEC in gastrointestinal surgery.

## 2. Materials and Methods

### 2.1. Medical Tactile Endosurgical Complex (MTEC)

**Figure 1:**
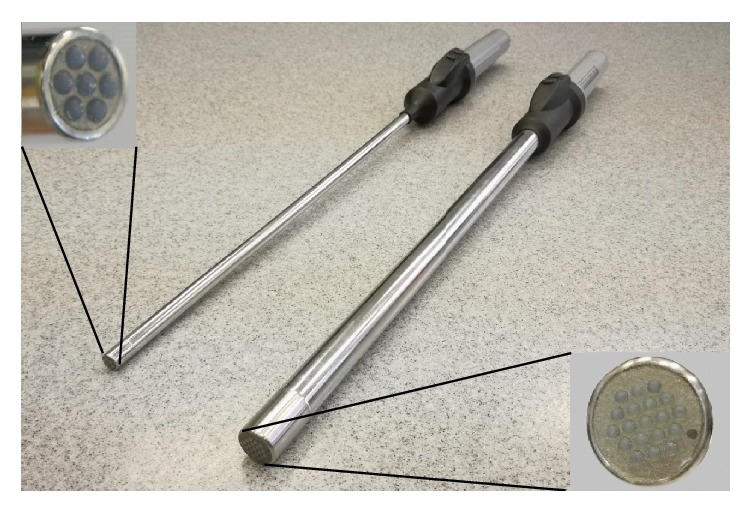
Tactile mechanoreceptors with 7 pressure sensors (left) and 19 pressure sensors (right).

The MTEC is a complex consisting of tactile mechanoreceptors, a computer, and an optional tactile display [16]. A tactile mechanoreceptor registers tactile data via pressure sensors located in its operating head and wirelessly transmits the results to a computer. A computer processes raw data [17], performs real-time visualisation of tactile images [16], and reproduces tactile images on a tactile display, where images can be perceived simply with the use of a finger. Two versions of tactile mechanoreceptors are available (Figure 1). The first one performs registration via 19 pressure sensors and has a 20 mm diameter. The second one has 7 pressure sensors and a diameter of 10 mm. The 20 mm version was designed primarily for utilisation in open surgery, while the 10 mm version was developed specifically for laparoscopic surgery.

**Figure 2:**
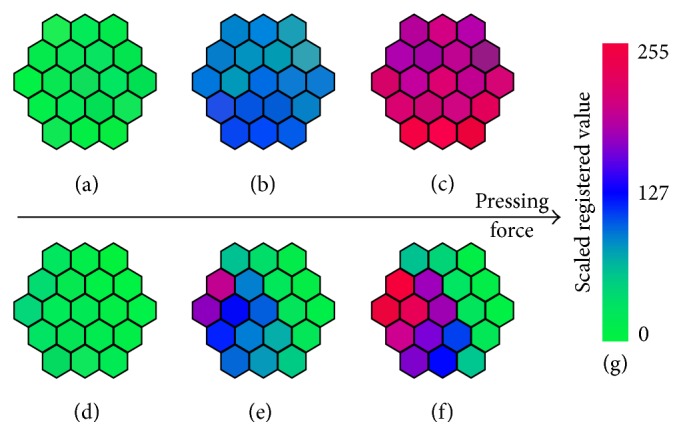
Visualisation of tactile frames registered during examination of a homogeneous area (a–c) and a heterogeneous area (d–f). Pressing force grows from left to right. Each hexagon corresponds to 1 pressure sensor. Registered values are scaled and colour-coded using a green-blue-red colour scale presented in panel (g).

**Figure 3:**
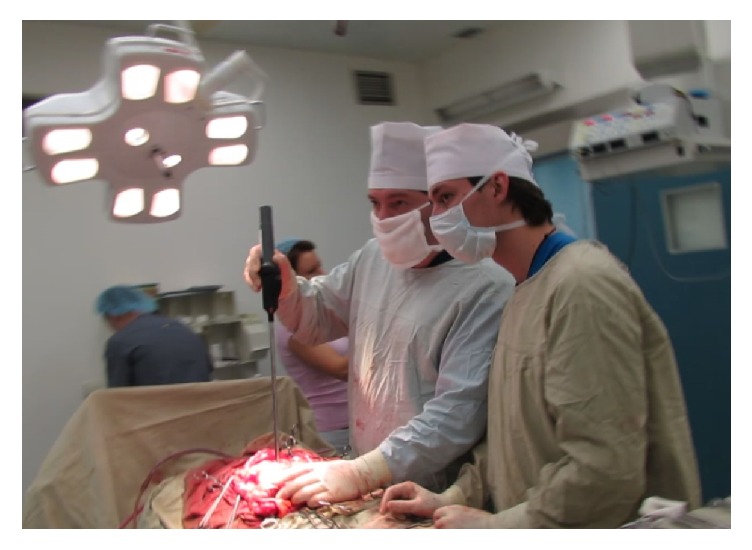
Instrumental mechanoreceptoric palpation in an open surgery.

Detection and localisation of tumours using MTEC are based on identification of lesion boundaries, which produce heterogeneous tactile images. A press on a homogeneous area results in homogeneous tactile frames, while a press on a boundary results in highly contrasting tactile frames (Figure 2; see also [16, Figures 2 and 3]).

### 2.2. Patients and Surgeries

The MTEC was tested in Clinical Hospital No. 31 (Moscow, Russia). The study was approved by the Ethics Committee of Clinical Hospital No. 31. Each patient signed informed consent form before surgery, which included having their depersonalised data used for medical scientific study/research and presented in any medical scientific/research paper. All surgeries were comprehensively registered.

From January 2013 to December 2015, 96 elective surgeries were performed, including 48 open surgeries, 43 laparoscopies, and 5 robot-assisted surgeries with the da Vinci™ robotic system (Intuitive Surgical, Sunnyvale, CA, USA). Patients—40 males and 56 females—were aged between 30 and 91 years, with an average age of 66.9 years (standard deviation: 12.4 years). Tumour localisation and type for the patients are summarised in Table 1. T stage distribution for malignant tumours is summarised in Table 2. Tumour long diameter data is summarised in Table 3.

All surgeries were performed by surgeons familiar with the technique of instrumental mechanoreceptoric palpation.

There were no side effects or postoperative complications related to instrumental mechanoreceptoric palpation.

### 2.3. Medical Tactile Endosurgical Complex (MTEC) Utilisation

In open surgeries, the 20 mm version of the tactile mechanoreceptor was used. For visually detectable tumours, instrumental mechanoreceptoric palpation was performed starting from the proximal end of the lesion towards the boundary. After the boundary was reached, instrumental mechanoreceptoric palpation was continued clockwise until complete localisation of the lesion was achieved. A contact angle between the tissue and the mechanoreceptor was kept maximally close to 90°. For visually undetectable tumours, instrumental mechanoreceptoric palpation started from the supposed site of localisation (determined by the preoperative diagnostic studies) and was carried out in the same manner. A surgeon perceived mechanoreceptorically registered tactile data through visualisation and reproduction on a tactile display and collated these data with the perception provided by conventional manual palpation.

In laparoscopic surgeries, the 10 mm version of the tactile mechanoreceptor was used. The tactile mechanoreceptor was inserted through a trocar port. Instrumental mechanoreceptoric palpation was carried out in the same way as was performed in open surgeries, but in laparoscopic surgeries larger deviations of a contact angle from 90° were unavoidable. In cases of intestine tumours, instrumental mechanoreceptoric palpation was followed by ultrasound examination performed with Flex Focus Ultrasound Machine (BK Medical, Herlev, Denmark).

In robot-assisted surgeries, the 10 mm version of the tactile mechanoreceptor was used. An assistant performed tactile examination via an additional port under the guidance of a surgeon [16]. A surgeon perceived tactile data through a tactile display. An assistant controlled the force applied to the tactile mechanoreceptor based on the visualisation of tactile images.

## 3. Results

### 3.1. Open Surgeries

The study began with testing MTEC in open surgeries (Figure 3). Open surgeries constituted the early stage of the learning curve for instrumental mechanoreceptoric palpation, and surgeons collated perception of tactile properties of tissues provided by instrumental and conventional palpation. In 44 out of 48 open surgeries (91.7%), lesions were visually detectable, and all these lesions were detectable by instrumental mechanoreceptoric palpation as well. Overall, lesions were detectable by instrumental mechanoreceptoric palpation in 47 out of 48 cases (97.9%).

The undetectable case was an adenocarcinoma of the rectosigmoid region, which was also impalpable by conventional palpation. Its precise localisation was determined using intraoperative colonoscopy.

The time of instrumental mechanoreceptoric palpation varied from 1 minute 30 seconds to 5 minutes 20 seconds, with an average of 3 minutes 12 seconds (details are presented in Table 4). Certain areas were inspected multiple times by surgeons in order to improve collation of instrumental and conventional palpation. The fastest examinations corresponded to lesions with stages T3 and T4 by TNM classification. More time was required for smaller lesions (stages T1 and T2). This difference is consistent with a similar difference for conventional manual palpation.

Analysis of intraoperative mechanoreceptoric palpation in open surgeries confirmed that a deviation of the contact angle between the tactile mechanoreceptor and the examined tissue from 90° essentially affects tactile images registered by MTEC and complicates their interpretation. As these deviations are unavoidable in laparoscopic surgeries, specific algorithms aimed at suppression of contact angle artifacts were implemented, tested, and included in the MTEC software [17]. Currently, MTEC software also includes additional preprocessing methods that compensate for the excessive force applied to a mechanoreceptor [17].

### 3.2. Laparoscopic Surgeries

**Figure 4:**
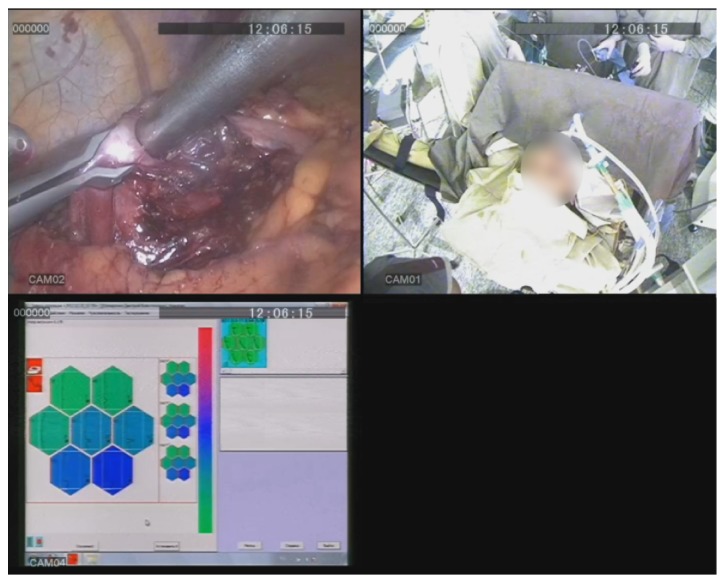
Instrumental mechanoreceptoric palpation of a stomach gastrointestinal stromal tumour (GIST) in laparoscopic surgery: simultaneously registered video streams from the external camera (CAM01), the laparoscopic camera (CAM02), and visualisation of tactile images (CAM04).

Lesion boundaries were clearly detected by instrumental mechanoreceptoric palpation in 41 out of 43 laparoscopic surgeries (95.3%) (Figure 4). The mean time of instrumental palpation—3 minutes 34 seconds—was slightly longer than that in open surgeries, but the difference was not significant. Detailed information about the time of instrumental palpation is presented in Table 4. In most of the laparoscopic surgeries, lesions were visually undetectable, despite the fact that in 5 cases, preoperative tattooing was performed.

In particular, in one case, visual localisation of the lesion was complicated despite preoperative tattooing. The patient had a polyp of the caecum opposite the ileocecal valve identified by colonoscopy. Instrumental mechanoreceptoric palpation took 5 minutes 15 seconds and provided correct localisation, which was then confirmed by intraoperative colonoscopy. The histological examination revealed tubular adenoma.

In the other case, multiple tubulovillous adenomas of the hepatic flexure colon were identified during colonoscopic examination and tattooed, but preoperative tattooing also appeared to be uninformative. Due to the soft consistency and close arrangement, instrumental mechanoreceptoric palpation provided localisation of only 1 lesion.

The remaining case in which a lesion could not be detected via instrumental mechanoreceptoric palpation was multiple neuroendocrine carcinomas of the ileum. Correct localisation in this case was achieved by intraoperative laparoscopic ultrasound examination.

### 3.3. Robot-Assisted Surgeries

In all 5 cases of instrumental mechanoreceptoric palpation performed in robot-assisted surgeries, lesions were correctly localised. The cases included 2 gastrectomies, 2 stomach resections, and a right hemicolectomy. These cases were described in [16], which is focused on using instrumental mechanoreceptoric palpation in robot-assisted surgery.

## 4. Discussion

The loss of the ability to palpate tissues and organs during laparoscopies essentially complicates localisation of visually undetectable tumours [7, 8, 18, 19]. At the same time, imprecise tumour localisation can lead to the removal of an incorrect segment of intestine [12]. Existing methods for tumour localisation still do not provide a 100% localisation rate and have specific limitations and possible complications. In up to 20% of cases, tumour locations identified by preoperative colonoscopy appear to be inconsistent with the intraoperative tumour site [9], and for preoperative tattooing, the incidence of invisible lesions reaches 15% [12, 13]. Intraoperative procedures, such as colonoscopy, gastroscopy, or ultrasonography, prolong surgical and anaesthesia time for patients. They also have their own challenges such as risk of splenic capsular tear, serosal tear of the colon or even perforation, and reduction of working space available to a surgeon due to bowel distention ([14, 20], [21, Ch. 3], and [22, Ch. 12]).

In this research, we evaluated the possibility of localisation of colorectal and gastric tumours via intraoperative instrumental mechanoreceptoric palpation performed using MTEC. This method is based on the fact that tactile properties of tumours are different from the tactile properties of normal adjacent tissues [16, 23] and serves as an analogue of conventional manual palpation. Our evaluation confirmed that instrumental mechanoreceptoric palpation is a simple and safe method with a short learning curve. It requires neither preoperative preparations nor presence of additional specialists at the time of surgery. The instrumental mechanoreceptoric palpation procedure is brief; furthermore, it can be used independently or in combination with other methods for lesion localisation. No side effects or postoperative complications related to this procedure were observed.

Localisation of lesions via instrumental mechanoreceptoric palpation performed using MTEC can be difficult in cases of small, soft lesions. This is consistent with the limitations for manual palpation [24–26].

Another limitation of this procedure is the requirement of only small deviations of the contact angle between the tactile mechanoreceptor and the examined tissue from 90°. This limitation was partially overcome by introducing special data processing methods into the MTEC software [17], but further improvements are desirable. They can be potentially achieved by optimisation of the construction of a tactile mechanoreceptor, particularly its operating head.

Localisation of gastrointestinal stromal tumours (GIST) via intraoperative instrumental mechanoreceptoric palpation was less apparent in comparison with other cases. It has been observed in both laparoscopic and robot-assisted surgeries [16]. In all GIST cases (including two laparoscopic and one robot-assisted surgeries) lesions were correctly localised, but due to palpatory indistinct boundaries and tactile heterogeneity of tumours it required more thorough instrumental palpation with very accurate application of MTEC mechanoreceptor.

## 5. Conclusion

Localisation of lesions by intraoperative instrumental mechanoreceptoric palpation performed using MTEC is a simple, safe, and reliable method which can be used either independently or in combination with other methods for tumour localisation. No side effects or postoperative complications related to instrumental mechanoreceptoric palpation were observed, and this procedure provided data sufficient for tumour localisation in 95.3% of laparoscopic surgeries.

## Figures and Tables

**Table 1:** Tumour localisation and type for patients involved in the study. GIST stands for gastrointestinal stromal tumour. “Other” tumour types included undifferentiated neoplasm (for gaster) and neuroendocrine carcinoma, hemangioma, fibroid polyp, and undifferentiated neoplasm (for intestine). Brackets contain numbers of open surgeries, laparoscopic surgeries, and robot-assisted surgeries, respectively.

*Type*	*Localisation*						*Total*
	Caecum and ascending colon	Sigmoid colon	Descending colon and transversum	Rectum	Gaster	Intestine	
Adenocarcinoma	25 (13/11/1)	25 (12/13/-)	16 (11/5/-)	10 (8/2/-)	3 (-/1/2)	—	*79 *(44/32/3)
Adenoma	6 (1/5/-)	1 (-/1/-)	1 (-/1/-)	—	—	—	*8 *(1/7/-)
GIST	—	—	—	—	3 (-/2/1)	1 (1/0/-)	*4 *(1/2/1)
Other	—	—	—	—	1 (-/-/1)	4 (2/2/-)	*5 *(2/2/1)

*Total number of patients*	*31 *(14/16/1)	*26 *(12/14/-)	*17 *(11/6/-)	*10 *(8/2/-)	*7 *(-/3/4)	*5 *(3/2/-)	*96 *(48/43/5)

**Table 2:** T stage distribution of malignant tumours for patients involved in the study. Brackets contain numbers of open surgeries, laparoscopic surgeries, and robot-assisted surgeries, respectively.

*T stage*	*Localisation*						*Total*
	Caecum and ascending colon	Sigmoid colon	Descending colon and transversum	Rectum	Gaster	Intestine	
T1	—	1 (1/-/-)	—	1 (1/-/-)	—	—	*2 *(2/-/-)
T2	2 (2/-/-)	2 (2/-/-)	1 (-/1/-)	—	4 (-/2/2)	2 (1/1/-)	*11 *(5/4/2)
T3	12 (3/8/1)	14 (3/11/-)	6 (4/2/-)	4 (2/2/-)	1 (-/-/1)	1 (1/-/-)	*38 *(13/23/2)
T4	11 (8/3/-)	8 (6/2/-)	9 (7/2/-)	5 (5/-/-)	2 (-/1/1)	—	*35 *(26/8/1)

**Table 3:** Tumour long diameter (cm) for patients involved in the study. The mean values are presented, along with the minimum and the maximum values (where applicable).

*Surgery type*	*Localisation*					
	Caecum and ascending colon	Sigmoid colon	Descending colon and transversum	Rectum	Gaster	Intestine
Open	6.14 (2–10)	4.58 (3–7)	7.77 (5–12)	6.38 (3.5–8)	—	5.00 (2–8)
Laparoscopic	3.44 (1–7)	4.00 (1–6)	3.75 (1–6)	3.50 (3-4)	3.67 (3–5)	1.40 (1.3–1.5)
Robot-assisted	5	—	—	—	5.88 (4–8.5)	—

**Table 4:** Duration of instrumental mechanoreceptoric palpation. The mean values are presented (minutes:seconds), along with the minimum and the maximum values (where applicable).

*Surgery type*	*Localisation*						*Total*
	Caecum and ascending colon	Sigmoid colon	Descending colon and transversum	Rectum	Gaster	Intestine	
Open	3:08 (1:45–4:30)	3:33 (2:00–4:45)	3:20 (1:30–5:20)	2:32 (1:35–4:05)	—	3:17 (2:20–4:15)	*3:12 *(1.30–5:20)
Laparoscopic	3:21 (1:45–5:30)	3:28 (1:50–5:05)	4:24 (2:55–6:40)	2:55 (2:40–3:10)	3:10 (2:30–4:15)	4:35 (3:40–5:30)	*3:34 *(1:45–6:40)
Robot-assisted	3:40	—	—	—	3:23 (2:35–4:30)	—	*3:26 *(2:35–4:30)
